# Eosinophils and other peripheral blood biomarkers in glioma grading: a preliminary study

**DOI:** 10.1186/s12883-019-1549-2

**Published:** 2019-12-05

**Authors:** Zhenxing Huang, Liang Wu, Zonggang Hou, Pengfei Zhang, Gen Li, Jian Xie

**Affiliations:** 1 Department of Neurosurgery, Beijing Tiantan Hospital, Capital Medical University, Beijing, China; 2 Department of International Medical Services, Beijing Tiantan Hospital, Capital Medical University, No.119 South Fourth Ring West Road, Fengtai District, Beijing, 100070 China

**Keywords:** Glioma, GBM, Eosinophils, Peripheral blood

## Abstract

**Background:**

Many peripheral blood biomarkers are associated with glioma grade, but eosinophils (Eo) are scarcely reported. This study assessed preoperative peripheral eosinophil levels and other peripheral biomarkers presented in prior literature, probing their associations and diagnostic value in the grading of glioma, including its most aggressive type, glioblastoma (GBM).

**Methods:**

Patients newly diagnosed with neuroepithelial tumors were included and divided into low-grade glioma (LGG)/high-grade glioma (HGG) groups and non-GBM/GBM groups separately. Preoperative peripheral biomarkers were collected, such as the counts of Eo, neutrophils (Neu), and lymphocytes (Ly), and values such as the eosinophil to lymphocyte ratio (ELR) and neutrophil to lymphocyte ratio (NLR) were calculated. Correlation analyses were also performed between these biomarkers and the groups. Receiver operating characteristic curves were utilized to assess the individual and joint diagnostic values of the biomarkers.

**Results:**

The HGG patients presented lower Eo and ELR values, which had negative correlations with glioma grade. The diagnostic efficiency of Eo and ELR could be enhanced when combined other biomarkers. In the non-GBM vs GBM analysis, GBM patients displayed reduced Eo and a negative correlation between Eo and a GBM diagnosis The combination of Eo and other biomarkers enhanced the diagnostic efficiency.

**Conclusions:**

A negative correlation between peripheral eosinophils and glioma grade was found in our study. Numerous cytokines derived from eosinophils could regulate the immune response and affect the tumor microenvironment; moreover, eosinophils may inhibit the tumorigenesis of glioma, which should be explored in the future and may enlighten some new paths for glioma therapy.

## Background

Gliomas, the most common tumor form in the central nervous system (CNS), can be divided into four histopathological grades (I - IV) according to the World Health Organization (WHO) classification. Generally, we refer to WHO grade I-II disease as low-grade glioma (LGG), while grade III-IV is regarded as high-grade glioma (HGG). Different grades of glioma are associated with a substantial disparity in prognosis. Patients with LGG can have relatively long periods of disease-free survival (DFS) [1], with median survival times of 2 years or even up to 12 years [2]. However, patients suffering from HGG, particularly the most malignant form, GBM, often have a poor prognosis, and the median survival time is only 16–18 months [3]. Gliomas have the characteristic of diffuse growth, and surgical resection currently plays a vital role in treatment. The principle of resection involves a balance with brain function preservation, and a maximum resection range is approved. Then, according to the histopathological tumor grade, postoperative radiotherapy and chemotherapy are usually indispensable [4]. For tumors that develop in eloquent areas, surgical resection is more challenging for neurosurgeons. Residual tumor (particularly HGG or GBM) could lead to a shorter survival period, but more aggressive resection may increase the opportunity for poor quality of life. Therefore, it is imperative to balance the resection range and function preservation, which reflects that the preoperative grading of glioma is significant for treatment.

At present, an invasive procedure (operation or biopsy) is believed to be the gold standard for diagnosing pathology. In the exploration of a noninvasive and inexpensive method, many studies have tried to find a credible approach to evaluate glioma grade preoperatively. Darbar et al. [5] tried to predict glioma grade via diffusion-weighted imaging (DWI). Diffusion tensor imaging (DTI) and diffusion kurtosis imaging (DKI) have also been linked to glioma grading in prior studies [6, 7]. However, the expensive cost, time-consuming nature and more expansive equipment requirements limit the usage of these advanced magnetic resonance imaging (MRI) techniques. Peripheral blood biomarkers, which have the merits of low costs and easy accessibility, have been reported to be associated with the prognosis and grade of many tumors, such as gastric carcinoma [8], lung cancer [9], renal cell carcinoma [10], and glioma [11–13]. In addition, biomarkers including the NLR, monocytes to lymphocytes ratio (MLR), platelets to lymphocytes (PLR), albumin (Alb), prognostic nutritional index (PNI), and systemic immune-inflammation index (SII) have been used frequently in glioma grading and prognosis in previous studies [11, 14].

Eosinophils, which are derived from myeloid progenitors, play vital roles in hypersensitivity, inflammation and antiparasitic reactions. However, many studies have found that the infiltration and degranulation of eosinophils in tumor tissue may indicate a positive prognosis for some solid tumors, such as colon cancer [15], nasopharynx cancer [16], bladder cancer and lung cancer [17], but eosinophils are scarcely reported in glioma. Considering that the grade of glioma dramatically affects prognosis, eosinophils may have a potential association with glioma grade. Hence, we designed this study to explore the relationship between the preoperative peripheral count of eosinophils and grade of glioma, especially GBM. We also embraced other peripheral blood biomarkers reported in previous studies and discussed their value in glioma grading.

## Methods

### Subject selection

A retrospective study was performed with a total of 360 patients derived from Beijing Tiantan Hospital between 2012 and 2017 and was approved by the Institutional Review Board of the Beijing Tiantan Hospital. The inclusion criteria were as follows: (1) newly diagnosed with neuroepithelial tumors by histopathological examination according to the WHO 2007 classification; (2) complete and accessible preoperative blood test; (3) no radiotherapy or chemotherapy before surgery; (4) no steroid or anti-inflammatory drugs used preoperatively; and (5) no autoimmune disease, hematological disease, active infection or tumors in other systems.

### Data selection and group criteria

The demographic data retrieved from medical records included age, sex, and pathology results. For blood biomarker data, we enrolled the counts of eosinophils (Eo), neutrophils (Neu), lymphocytes (Ly), monocytes (Mono) and platelets (PLT) as well as the levels of albumin (Alb), globulin (Glb), and fibrinogen (Fbg) measured during the routine preoperative blood test, which we defined as the biomarkers-original (Biomarkers-ori). The NLR, MLR, PLR, ELR, PNI, and SII, which we denoted as the biomarkers-calculated (Biomarkers-cal), were calculated from the Biomarkers-ori. The calculation methods are described below.

- ELR = count of eosinophils/count of lymphocytes
- NLR = count of neutrophils/count of lymphocytes
- MLR = count of monocytes/count of lymphocytes
- PLR = count of platelets/count of lymphocytes
- PNI = Alb level + count of lymphocytes × 5
- SII = count of platelets × the NLR

Patients diagnosed with WHO I-II grade disease were defined as LGG, while those diagnosed with WHO III-IV grade disease were defined as HGG. Meanwhile, the division of GBM vs non-GBM patients was also discussed in this study.

Since most of our cases were retrieved before 2016, the detailed molecular pathology data were incomplete or even absent. To minimize the bias in our study, we recorded the patients’ molecular pathology data, if any, and hypothesized that if the different of molecular pathology statuses within the same glioma grade did not influence the Eo or ELR values, which we mainly discuss in present study, then the variation tendencies of Eo and ELR between the groups in our study could reflect the real trend. After we checked the existing molecular pathology data, we selected the following relatively complete datasets that could be competent for statistical analysis: IDH1, MGMT and 1p/19q; and statistical analysis was performed among grade 2, grade 3 and grade 4, respectively. Due to the rarity of cases and the lack of molecular pathology data for most cases, a group for grade 1 was not included in this part.

### Statistical methods

The categorical variables are presented as percentages. The continuous variables were tested for the adaption of a Gaussian distribution via the Kolmogorov-Smirnov test first, which showed that none of the continuous variables except for the PNI conformed to a Gaussian distribution. Thus, an average ± SD is presented for the PNI, and a median with 25th and 75th percentiles is presented for the remaining biomarkers.

A Mann-Whitney test, independent t test or chi-square test was applied to compare different types of variables between groups. Correlations between biomarkers and groups were analyzed by the Spearman correlation coefficient test. A receiver operating characteristic (ROC) curve analysis was also performed to acquire the area under the curve (AUC) value that evaluated the diagnostic efficiency, as well as cut-offs for biomarkers-ori. For the molecular pathology analysis, due to the small size of the sample, we utilized Mann-Whitney test and calculated the exact *P* value. SPSS 24.0 and GraphPad Prism 8 were used for the statistical procedures and graphics generation. A *P* value less than 0.05 in a 2-tailed test was considered statistically significant.

## Results

### Demographics of the study patients

A total of 360 patients were enrolled in this study, including 224 males and 136 females. In the LGG vs HGG group analysis, 165 patients were diagnosed with LGG, and 195 patients were diagnosed with HGG. In the non-GBM vs GBM group analysis, the numbers of patients with GBM or non-GBM disease were 106 and 254, respectively. More detailed demographic information is presented in Table 1.

**Table 1:** Demographic characteristics of the study patients

Parameter	Median/Mean (IQR/SD)	Patient number	Percentage
Sex			
Male	–	224	62.2%
Female	–	136	37.8%
Glioma grade			
LGG	–	165	45.8%
HGG	–	195	54.2%
GBM status			
Non-GBM		254	70.6%
GBM		106	29.4%
Age (years)	41 (33–50)		
Eo (×10^9^/L)	0.07 (0.04–0.12)		
Neu (× 10^9^/L)	4.23 (3.31–5.44)		
Mono (× 10^9^/L)	0.39 (0.30–0.50)		
Ly (× 10^9^/L)	1.83 (1.53–2.30)		
PLT (× 10^9^/L)	221 (187.25–265.00)		
ELR	0.039 (0.022–0.065)		
NLR	2.223 (1.629–3.189)		
MLR	0.21 (0.16–0.26)		
PLR	119.383 (93.148–150.950)		
Alb (g/L)	46.300 (44.200–48.000)		
Glb (g/L)	26.800 (24.300–29.000)		
Fbg (g/L)	2.665 (2.250–3.110)		
PNI	55.879 ± 4.680		
SII	526.938 (344.974–719.950)		

### Evaluation of potential influences on molecular pathology status

The results showed the molecular pathology factors, except for MGMT, could impact the ELR in grade 2 glioma (*P* = 0.04), but none influenced Eo and ELR (Table 2, Table 3, Table 4). Among grade 2 gliomas, MGMT promoter methylation was associated with a higher ELR. These results exhibit that the molecular status has a slight impact on Eo and ELR.

**Table 2:** Potential influences on Eo and ELR caused by IDH1 mutation within glioma grade

	Grade2			Grade3			GBM		
	Wild type (*n* = 2)	Mutation type (*n* = 18)	*P*	Wild type (*n* = 3)	Mutation type (*n* = 19)	*P*	Wild type (*n* = 11)	Mutation type (*n* = 12)	*P*
Median of Eo	0.125	0.06	0.279	0.1	0.06	0.333	0.06	0.055	0.915
Median of ELR	0.06	0.039	0.38	0.027	0.033	0.787	0.04	0.026	0.577

**Table 3:** Potential influences on Eo and ELR caused by MGMT promoter methylation status within glioma grade

	Grade2			Grade3			GBM		
	MGMT promoter methylation (*n* = 73)	non-MGMT promoter methylation (*n* = 23)	*P*	MGMT promoter methylation (*n* = 41)	non-MGMT promoter methylation (*n* = 15)	*P*	MGMT promoter methylation (*n* = 53)	non-MGMT promoter methylation (*n* = 22)	*P*
Median of Eo	0.08	0.05	0.076	0.07	0.08	0.481	0.06	0.06	0.864
Median of ELR	0.045	0.025	0.04	0.039	0.037	0.519	0.036	0.03	0.447

**Table 4:** Potential influences on Eo and ELR caused by 1p/19q status within glioma grade

	Grade2			Grade3			GBM		
	1p/19q codeletion (*n* = 54)	non-1p/19q codeletion (*n* = 56)	*P*	1p/19q codeletion (*n* = 26)	non-1p/19q codeletion (*n* = 31)	*P*	1p/19q codeletion (*n* = 2)	non-1p/19q codeletion (*n* = 64)	*P*
Median of Eo	0.08	0.075	0.724	0.05	0.07	0.646	0.185	0.06	0.066
Median of ELR	0.045	0.042	0.767	0.035	0.034	0.91	0.076	0.03	0.146

## LGG vs HGG

### Demographic characteristics

In the LGG patient population, there were 105 males and 60 females, and the median age was 40 (31–45) years. The HGG patient population included 119 males and 76 females with a median age of 45 (34–53) years. More detailed information is provided in Table 5.

**Table 5:** Demographic data and parameter comparisons between the LGG and HGG groups

Parameter	LGG (*n* = 165)	HGG (*n* = 195)	*P* value
Sex			0.097
Male	105 (63.6%)	119 (61.0%)	
Female	60 (36.4%)	76 (39.0%)	
Age (years)	40 (31–45)	45 (34–53)	< 0.001
Eo (×10^9^/L)	0.08 (0.05–0.15)	0.06 (0.04–0.10)	0.006
Neu (× 10^9^/L)	4.060 (3.240–4.960)	4.530 (3.430–6.260)	0.002
Mono (× 10^9^/L)	0.370 (0.290–0.460)	0.410 (0.320–0.560)	0.004
Ly (× 10^9^/L)	1.860 (1.570–2.345)	1.800 (1.480–2.280)	0.072
PLT (× 10^9^/L)	221 (190.00–268.50)	222.00 (182.00–264.00)	0.651
ELR	0.042 (0.025–0.069)	0.036 (0.020–0.061)	0.027
NLR	1.966 (1.524–2.743)	2.394 (1.776–3.427)	< 0.001
MLR	0.191 (0.157–0.242)	0.225 (0.170–0.289)	< 0.001
PLR	118.63 (89.500–148.33)	120.43 (96.060–151.50)	0.314
Alb (g/L)	46.700 (44.650–48.550)	46.000 (43.900–47.800)	0.013
Glb (g/L)	26.300 (24.250–29.000)	27.100 (24.300–29.000)	0.290
Fbg (g/L)	2.470 (2.205–2.880)	2.820 (2.340–3.210)	< 0.001
PNI	56.457 ± 4.270	55.390 ± 4.959	0.031
SII	476.41 (317.83–656.50)	550.00 (378.82–797.94)	0.003

### Parameter comparisons

**Fig. 1:**
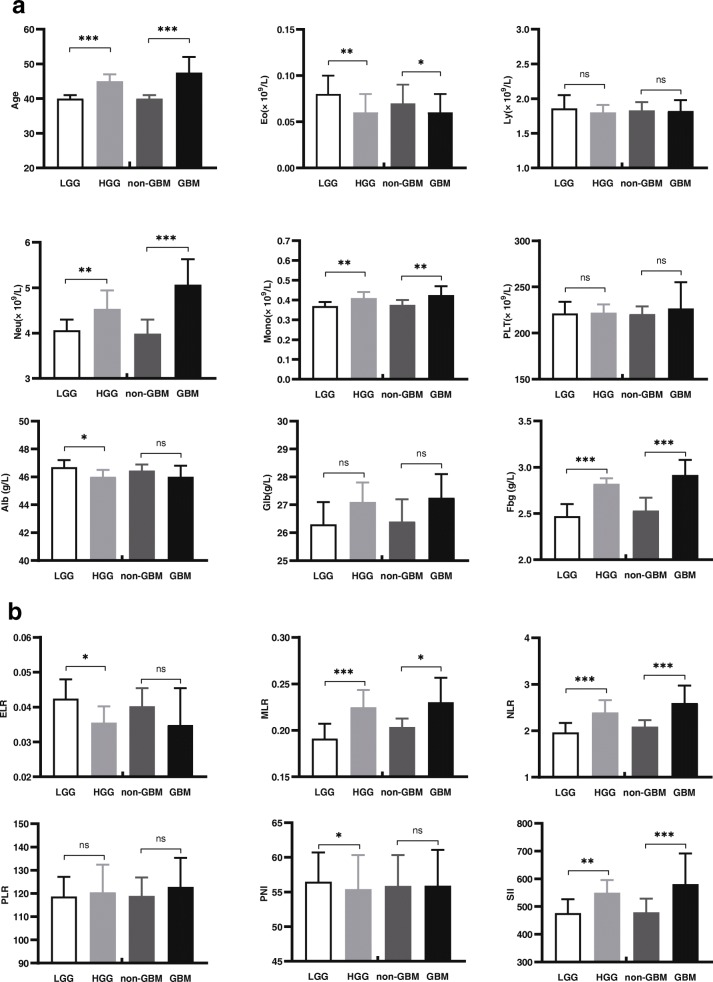
Histograms with error bars of the comparisons in LGG vs HGG and non-GBM vs GBM for the parameters (**a**) age and preoperative peripheral blood biomarkers-ori, and (**b**) preoperative peripheral blood biomarkers-cal, * *P* < 0.05, ** *P* < 0.01, *** *P* < 0.001, ns stands for not significant.

Based on our data, we found that the HGG patients had an older age; higher Neu, Mono, NLR, MLR, SII and Fbg values; and lower median Eo, ELR, Alb and PNI values (each *P* < 0.05). The Ly was decreased in the HGG patients, but the difference was not statistically significant (*P* = 0.072). The comparisons are shown in Table 5 and Fig. 1.

### Correlations between parameters and glioma grade

Next, we enrolled significant parameters in a Spearman correlation analysis and found that age, the Neu, the Mono, the NLR, the MLR, Fbg levels and the SII had positive correlations with HGG, while the Eo, the ELR, Alb levels and the PNI displayed negative correlations (Table 6).

**Table 6:** Spearman correlation analysis for the LGG vs HGG patient stratification

Parameter	LGG vs HGG	
	Correlation (r)	*P* value
Age	0.234	< 0.001
Eo	−0.146	0.005
Neu	0.167	0.002
Mono	0.153	0.004
ELR	−0.117	0.027
NLR	0.209	< 0.001
MLR	0.199	< 0.001
Alb	−0.132	0.012
Fbg	0.202	< 0.001
PNI	−0.133	0.011
SII	0.158	0.003

### Diagnostic efficiency of the Eo, the ELR and other parameters for HGG and LGG

**Fig. 2:**
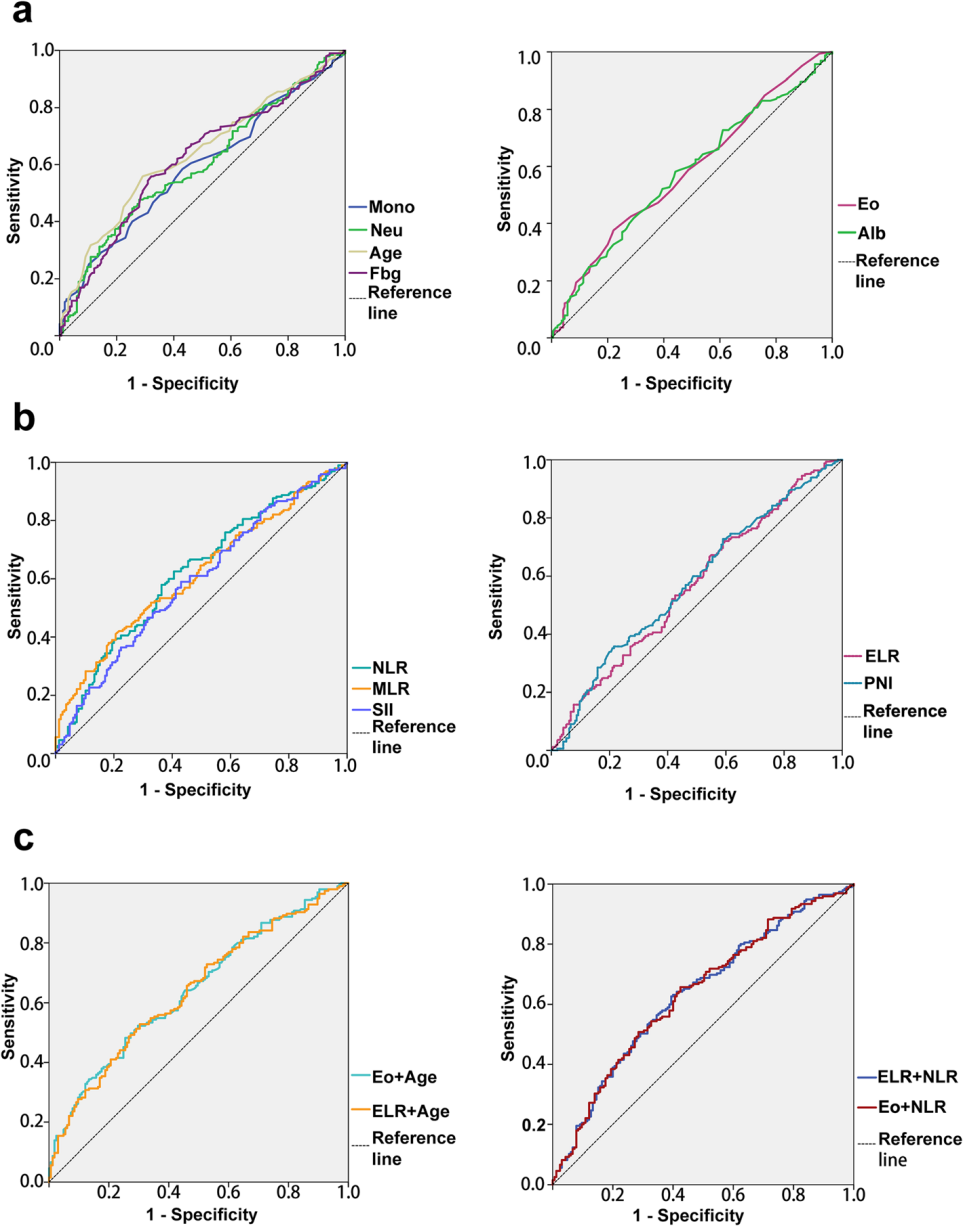
Diagnostic value of the following parameters when patients were stratified as LGG or HGG: **a** biomarkers-ori and age, **b** biomarkers-cal, and **c** combinations of the Eo or ELR with age or NLR

A ROC analysis was utilized to evaluate the cut-off and diagnostic values, as shown in Table 7 and Fig. 2. Since the Eo, ELR, Alb and PNI showed a negative trend with increasing grade, patients with corresponding values greater than the cut-offs tended to have LGG. Additionally, when age, Neu, Mono, NLR, MLR, SII and Fbg were greater than the corresponding cut-offs, the patients could be considered to have HGG.

**Table 7:** Diagnostic efficiency of parameters for distinguishing LGG and HGG

Parameter	Cut-off value	AUC (95% CI)
Age (years)	44	0.636 (0.579–0.693)
Eo (×10^9^/L)	0.105	0.585 (0.526–0.644)
Neu (×10^9^/L)	4.785	0.597 (0.538–0.655)
Mono (×10^9^/L)	0.385	0.589 (0.530–0.647)
ELR	0.027	0.568 (0.509–0.627)
NLR	2.180	0.621 (0.563–0.679)
MLR	0.251	0.616 (0.558–0.673)
Alb (g/L)	46.250	0.576 (0.517–0.636)
Fbg (g/L)	2.745	0.617 (0.559–0.675)
PNI	58.450	0.577 (0.518–0.636)
SII	506.05	0.591 (0.533–0.650)
Age + Eo	–	0.642 (0.586–0.699)
Age + ELR	–	0.639 (0.582–0.696)
NLR + Eo	–	0.634 (0.577–0.692)
NLR + ELR	–	0.634 (0.577–0.692)

Age had the highest AUC (0.636), and the NLR ranked second with an AUC of 0.621. The AUCs of eosinophil-associated parameters were 0.585 for the Eo and 0.568 for the ELR, which we combined with age and the NLR to elevate the diagnostic value. With an AUC of 0.642, age + Eo exhibited the highest diagnostic value.

## Non-GBM vs GBM

### Demographic characteristics

A total of 254 patients were diagnosed with non-GBM, including 160 males and 94 females, and their median age was 40 years old. Among the 106 GBM patients, there were 64 males and 42 females, and the median age was 47.5 years old. Detailed data are presented in Table 8.

**Table 8:** Demographic data and parameter comparisons between the non-GBM and GBM groups

	non-GBM (*n* = 254)	GBM (*n* = 106)	*P* value
Sex			< 0.001
Male	160 (63.0%)	64 (60.4%)	
Female	94 (37.0%)	42 (39.6%)	
Age (years)	40 (32–47)	47.5 (36–56)	< 0.001
Eo (× 10^9^/L)	0.07 (0.04–0.12)	0.06 (0.03–0.10)	0.036
Neu (× 10^9^/L)	3.985 (3.240–4.960)	5.065 (3.780–6.978)	< 0.001
Mono (× 10^9^/L)	0.375 (0.300–0.472)	0.425 (0.318–0.583)	0.005
Ly (× 10^9^/L)	1.83 (1.528–2.290)	1.82 (1.530–2.338)	0.986
PLT (× 10^9^/L)	220.50 (185.75–263.00)	226.50 (192.00–272.00)	0.233
ELR	0.040 (0.024–0.066)	0.035 (0.017–0.06)	0.06
NLR	2.088 (1.575–2.844)	2.595 (1.878–3.553)	< 0.001
MLR	0.204 (0.161–0.257)	0.230 (0.165–0.297)	0.028
PLR	118.84 (92.151–148.54)	122.76 (96.400–157.65)	0.528
Alb (g/L)	46.45 (44.40–48.20)	46.000 (43.975–47.650)	0.226
Glb (g/L)	26.400 (24.300–28.800)	27.250 (24.300–29.625)	0.207
PNI	55.874 ± 4.467	55.892 ± 5.178	0.974
SII	479.46 (329.22–669.02)	580.84 (412.64–905.45)	< 0.001
Fbg (g/L)	2.530 (2.220–2.960)	2.915 (2.420–3.330)	< 0.001

### Parameter comparisons

Through statistical analysis, we found that males were more susceptible to GBM, and older age and higher Neu, Mono, NLR, MLR, SII and Fbg values were also present in the GBM patients(*P* < 0.05). The Eo was reduced in the patients with GBM(*P* < 0.05). The ELR was decreased in the patients with GBM compared with the patients with non-GBM disease, and this difference almost reached statistical significance, with a *p* value of 0.06; similarly, the Ly was decreased but not significantly different. Detailed information is presented in Table 8 and Fig. 1.

### Correlations between parameters and GBM

Similarly, we included significant parameters in a correlation analysis and found that only the Eo presented a negative correlation with GBM, while the rest of the parameters showed positive correlations (Table 9).

**Table 9:** Spearman correlation analysis for the non-GBM vs GBM patient stratification

Parameter	non-GBM vs GBM	
	Correlation (r)	*P* value
Age	0.245	< 0.001
Eo	−0.110	0.036
Neu	0.247	< 0.001
Mono	0.147	0.005
NLR	0.217	< 0.001
MLR	0.116	0.028
Fbg	0.217	< 0.001
SII	0.203	< 0.001

### Diagnostic efficiency of the Eo and other parameters in GBM

**Fig. 3:**
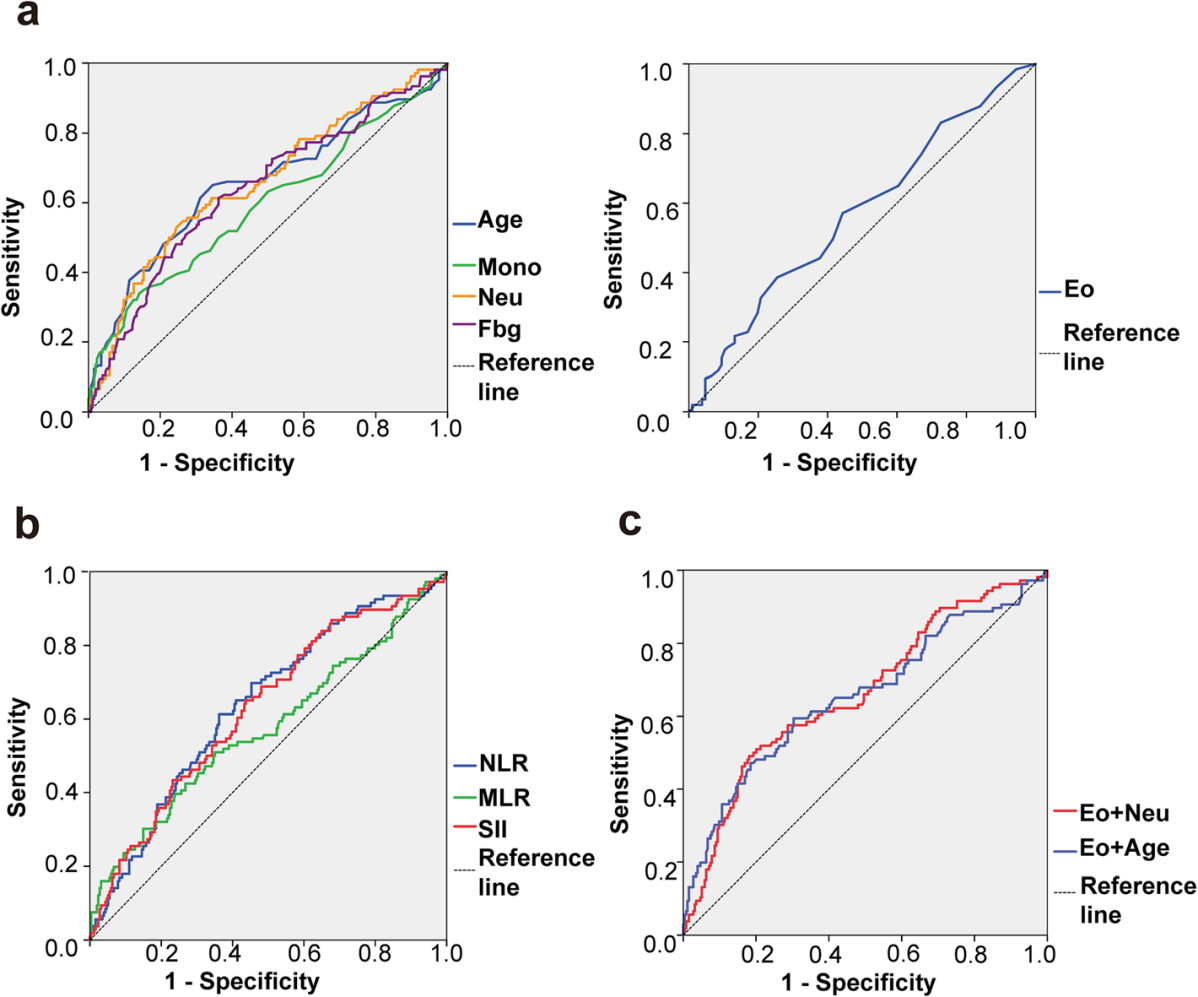
Diagnostic value of the following parameters when patients were stratified as non-GBM or GBM: **a** biomarkers-ori and age, **b** biomarkers-cal, and **c** the Eo combined with age or the Neu

The significant parameters were also analyzed using a ROC analysis (Table 10 and Fig. 3), and we found that the Neu showed the highest AUC (0.656), with age showing the next highest (0.655). The Eo achieved an AUC of 0.57, which could be increased when the Eo was combined with the Neu or age. The highest AUC was 0.663 for Eo + Neu.

**Table 10:** Diagnostic value of parameters for distinguishing non-GBM glioma from GBM

Parameter	Cut-off value	AUC (95% CI)
Age (years)	44	0.655 (0.588–0.722)
Eo (×10^9^/L)	0.095	0.570 (0.505–0.634)
Neu (×10^9^/L)	4.975	0.656 (0.524–0.661)
Mono (×10^9^/L)	0.525	0.593 (0.524–0.772)
NLR	2.369	0.638 (0.575–0.700)
MLR	2.229	0.573 (0.505–0.642)
Fbg (g/L)	2.760	0.637 (0.573–0.702)
SII	526.94	0.629 (0.565–0.692)
Eo + Neu	–	0.663 (0.600–0.726)
Eo + Age	–	0.655 (0.589–0.721)

Regarding the cut-offs, because GBM tends to have a lower Eo, an Eo value less than 0.095 ×  10^9^/L could suggest a GBM, while an age, Neu, Mono, NLR, MLR, SII and Fbg greater than the corresponding cut-offs would lean towards a GBM.

## Discussion

Eosinophils are well understood to be associated with atopic diseases and allergic and antiparasitic reactions. Furthermore, eosinophils, as an innate immune cell, are also associated with many complicated immunoreactions, including tumorigenesis. As previously reported, the role that eosinophils play in tumorigenesis remains controversial. However, eosinophils are believed to inhibit the growth of tumors and are associated with a positive prognosis in some solid tumors [18, 19], such as colon cancer [15] and nasopharyngeal cancer [16]. A study by Costello et al. [17] also showed a good prognosis when eosinophils infiltrated laryngeal cancer, bladder carcinoma and lung cancer, but eosinophil infiltration in Hodgkin lymphoma might lead to a poor prognosis [20].

Interestingly, from an epidemiological perspective, as the level of eosinophils increases in patients with atopic diseases [18], the risk of glioma decreases [21]. A recent population-based study showed a negative correlation between asthma and the risk of glioma, and active asthma had a more inverse association with the risk of glioma than inactive asthma [22]. This phenomenon shows that eosinophils might be a protective factor for glioma growth.

The mechanism connecting eosinophils with tumorigenesis remains unclear. A recent study reported that dipeptidyl peptidase 4 inhibitor could recruit eosinophils into the tumor tissue and inhibit its growth [23], which could be a potential explanation for the role of eosinophils in anti-tumor reactions. Holl et al. found that circulating eosinophils were absent and the level of circulating neutrophils was elevated in HGG patients compared with melanoma and breast cancer patients, and they also found that there was less lymphocyte infiltration in the tumor tissue [24]. This also laterally reflected the complex and unique immunoreactions in glioma development, which also could influence the peripheral immune cells. Additionally, a review of previous studies suggests that the probable mechanism might be related to the influence of granule proteins, cytokines and chemokines secreted by eosinophils on the immune system.

A degranulation phenomenon that releases eosinophil granule proteins, including major basic protein (MBP), eosinophil-derived neurotoxin (EDN), and eosinophil cationic protein (ECP) [25], is often observed with eosinophils located in tumor tissue [26]. MBP leads to tumor cell cytotoxicity via damage to the tumor cell lipid bilayer [27]. In a GBM model, EDN, a ligand of toll-like receptor-2 (TLR2) that can induce immune cells to infiltrate tumor tissue and inhibit tumor growth [28, 29], may be favorable for inhibiting GBM cells [21]. Boix et al. [30] considered that ECP could regulate the permeability of the cell membrane to have a cytotoxic effect. The role of EPO in the antitumor response remains to be discussed. Nathan et al. [31] reported that EPO, in cooperation with macrophages, can kill tumor cells and catalyze peroxidative oxidation, leading to either DNA mutations or effects on tumor cell aging and apoptosis [21, 32], which may promote tumorigenesis.

Eosinophils can also secrete many cytokines that influence the immune system. Th1-associated cytokines secreted by eosinophils (e.g., IL-8, TGF-α, and IFN-γ) may contribute to the antitumor response [33], while Th2-associated cytokines (e.g., IL-5) released by eosinophils do not enhance the activity of immunocytes and are linked to a worse prognosis [33, 34]. However, it is not clear which type of cytokine is secreted by eosinophils in the tumor microenvironment. Carretero et al. [35] reported that some cytokines secreted by eosinophils can contribute to not only the recruitment of CD8^+^ T cells into tumor tissue but also the normalization of tumor vasculature and polarization of macrophages into the M1 type, which is related to an improved prognosis. Furthermore, a mouse model study found that elevated peripheral eosinophil numbers lead to massive tumor tissue infiltration by eosinophils and tumorigenesis inhibition. In eosinophil-deficient mice, the incidence of tumors is increased with the total absence of eosinophils [36].

Recently, a lncRNA called eosinophil granule ontogeny transcript (EGOT) has been related to the antitumor response of eosinophils. EGOT is involved in eosinophil development and expressed in mature eosinophils [37]. Xu et al. [38] found that low expression of EGOT in breast cancer leads to an increased tumor volume, increased lymph node metastasis and a worse prognosis.

EGOT is also connected with glioma. Wu et al. [39] reported that the expression of EGOT in glioma tissue is lower than that in nontumor tissue. Moreover, the expression level of EGOT varies among different glioma cell lines, with the lowest level in the U251 and U87 cell lines, which are believed to be the most aggressive type. This phenomenon might be because the expression of EGOT can arrest the cell cycle in the G0/G1 phase [39] and reduce glioma cell proliferation.

Although the role of eosinophils in tumorigenesis is not quite clear, prior studies have reported that eosinophils relate to prognosis in some solid tumors, including gliomas. In addition, the prognosis of glioma is associated with the tumor grade. Therefore, all of these findings suggest some latent relation between eosinophils and the grade of glioma, which we mainly support. Finally, the anti-tumor response in eosinophils is associated with both cytotoxicity substances in glioma cells and the regulation of other immune cell infiltrates into glioma, and these dual tumor-inducing and tumor-suppressing effects must be orchestrated between the tumor and normal brain [40].

In our present study, regarding the parameters of the Biomarkers-ori, we mainly found that the Eo value was lower in patients with HGG or GBM than in their respective counterparts. The Eo value also exhibited negative correlations with HGG and GBM. Considering the literature reported above, these results could also explain why most HGG and GBM patients experienced rapid tumor progression and that eosinophils have a tumor suppressing response.

In addition to the Eo, the Neu and Mono were also found to be higher in the patients with HGG or GBM than in their respective counterparts. A decreased Ly trend also emerged but was not statistically significant. The characteristic variations were similar to those in previous studies. A multicenter study performed by Zheng et al. [11] reported that the Neu and Mono increased with increasing glioma grading, while the Ly decreased. Weng et al. [12] concluded the same results. This changing trend might depend on the tumor microenvironment inducing an abnormal inflammatory state for glioma [40], which leads to an elevated Neu and a decreased Ly [41, 42]. Some cytokines (e.g., IL-10 and IL-12) secreted by GBM cells inhibit the adaptive immune response [43], which may contribute to the decrease in lymphocyte numbers. According to a recent report, HGG can also sequester T cells in the bone marrow, which may give rise to the lower Ly in the peripheral blood [44]. Domenis et al. [45] showed that exosomes derived from gliomas may promote the maturation of monocytes that can suppress the effector activities of T cells, which may relate to the elevated monocyte numbers.

The changing tendencies of the Biomarkers-cal, such as elevated NLR and MLR in HGG and GBM patients, were calculated from the Biomarkers-ori. Thus, the variations in the Biomarkers-cal trends were consistent with the variations in the Biomarkers-ori that were described above. Weng et al. [12] found that a higher NLR is associated with HGG and that an elevated NLR in GBM patients indicates a poor prognosis. Zheng et al. [11] also reported that an increased NLR and a decreased LMR indicate a higher grade of glioma. In this study, we reached a similar conclusion.

Interestingly, the ELR, a newly built parameter in our study, was lower in our HGG patients than in our LGG patients (*P* < 0.05) and had a decreased value in the GBM patients, nearly reaching statistical significance with *P* = 0.06. Moreover, among the constituent parts of the ELR, the Eo decreased in our HGG and GBM patients (*P* < 0.05), and the Ly also revealed a decreasing trend, which was not significant. We could therefore conclude that the Eo fell more rapidly than the Ly as the glioma grade increased. However, there are few reports in the literature that explain why the Eo would decrease more in HGG and GBM. Nevertheless, eosinophils act as innate immune cells and can secrete numerous cytokines related to the immune response, which is strongly regulated by the state of the tumor microenvironment [46]. Hence, whether HGG or GBM could inhibit the development of eosinophils and further interfere with immunity may be a new consideration for glioma immunotherapy.

In addition, in grade 2 glioma patients, we also observed that the ELR was higher in tumors with MGMT promoter methylation than in those without methylation. This result showed that an elevated ELR may lead to a better prognosis and could be considered as a positive (protective) factor, which was consistent with the conclusion mentioned above. However, the associations among eosinophils, lymphocytes and MGMT remain unknown.

The Alb level, PNI and SII are associated with the state of nutrition and immunity and are linked with glioma grade and prognosis. A decreased PNI might reflect a worse nutrition state and relate to a higher grade of disease and a poor prognosis [11, 47]. Calculated from the PLT, Neu and Ly, all of which might relate to the proliferation and differentiation of glioma [4], the an elevated SII appears to be linked with higher grade glioma and a worse prognosis [4, 48, 49]. Furthermore, we also found that an increased Fbg level was correlated with higher grade glioma, which might result from Fbg contributing to tumor angiogenesis and metastasis [50].

Based on the data in our study and ROC curve analysis, the Eo and ELR have the ability to predict glioma grade, and Eo exhibited some value in predicting GBM as well. However, the AUCs of these parameters were not higher than those of previous parameters (e.g., the NLR and MLR), which might result from the complexity of eosinophil roles in tumorigenesis. Thus, a combination could enhance their predictive value, similar to the findings in the study by Zheng et al. [11]. Hence, we recommend combining these parameters for clinical application. Furthermore, we also found that older people and males were more vulnerable to GBM, which is consistent with an epidemiological study of CNS tumors in the United States [51].

This study has several limitations. First, the missing molecular pathology data should be pointed out, especially as these data are associated with the prognosis. Given this circumstance, we did not perform a survival analysis between prognosis and eosinophils. However, we tested the impact of the molecular pathology factors, and they had no impact on the eosinophil-associated parameters, which were the main focus of the present study. Second, the study was retrospective in nature and only peripheral eosinophils were included, so more research is needed regarding eosinophils located in the tissue of the tumor periphery.

## Conclusion

In this study, similar to the reduced lymphocytes in glioma patients, a lower level of peripheral eosinophils was associated with a higher grade of glioma. As previously reported peripheral biomarkers, eosinophils were valuable in glioma grading and GBM diagnosis. The combination of eosinophils with other parameters would enhance the overall diagnostic efficiency. Hence, eosinophils can also inhibit the tumorigenesis of glioma; the role of eosinophils in the natural course of glioma needs to be determined in the future, which may enlighten some new paths for glioma therapy.

## Data Availability

The datasets used and/or analyzed during the current study available from the corresponding author on reasonable request.

## Abbreviations

Alb: Albumin
ECP: Eosinophil cationic protein
EDN: Eosinophil-derived neurotoxin
EGOT: Eosinophil granule ontogeny transcript
ELR: Eosinophils to Lymphocytes ratio
Eo: Eosinophils
Fbg: Fibrinogen
GBM: Glioblastoma
Glb: Globulin
HGG: High-grade glioma
LGG: Low-grade glioma
Ly: Lymphocytes
MBP: Major basic protein
MLR: Monocytes to Lymphocytes ratio
Mono: Monocytes
Neu: Neutrophils
NLR: Neutrophils to Lymphocytes ratio
PLR: Platelets to Lymphocytes
PLT: Platelets
PNI: Prognostic nutritional index
SII: Systemic immune-inflammation index

## References

[1] Aaronson NK (2011). Compromised health-related quality of life in patients with low-grade glioma. J Clin Oncol.

[2] Schiff D (2017). Low-grade Gliomas. Continuum (Minneap Minn).

[3] Nayak L, Reardon DA (2017). High-grade Gliomas. Continuum (Minneap Minn).

[4] Liang R (2018). Significance of systemic immune-inflammation index in the differential diagnosis of high- and low-grade gliomas. Clin Neurol Neurosurg.

[5] Darbar A (2018). Use of preoperative apparent diffusion coefficients to predict brain tumor grade. Cureus.

[6] Jiang L (2017). Analysis of DTI-derived tensor metrics in differential diagnosis between low-grade and high-grade Gliomas. Front Aging Neurosci.

[7] Qi XX (2018). Histogram analysis of diffusion kurtosis imaging derived maps may distinguish between low and high grade gliomas before surgery. Eur Radiol.

[8] Ubukata H (2010). Evaluations of interferon-gamma/interleukin-4 ratio and neutrophil/lymphocyte ratio as prognostic indicators in gastric cancer patients. J Surg Oncol.

[9] Sarraf KM (2009). Neutrophil/lymphocyte ratio and its association with survival after complete resection in non-small cell lung cancer. J Thorac Cardiovasc Surg.

[10] Hu H (2017). Prognostic value of preoperative NLR, dNLR, PLR and CRP in surgical renal cell carcinoma patients. World J Urol.

[11] Zheng SH (2018). Diagnostic value of preoperative inflammatory markers in patients with glioma: a multicenter cohort study. J Neurosurg.

[12] Weng W (2018). Preoperative neutrophil-lymphocyte ratio correlated with glioma grading and glioblastoma survival. Neurol Res.

[13] Wang PF (2018). Preoperative changes in hematological markers and predictors of Glioma grade and survival. Front Pharmacol.

[14] Xu W (2018). Sex-dependent association of preoperative hematologic markers with glioma grade and progression. J Neuro-Oncol.

[15] Pretlow TP (1983). Eosinophil infiltration of human colonic carcinomas as a prognostic indicator. Cancer Res.

[16] Fujii M (2002). Significance of epidermal growth factor receptor and tumor associated tissue eosinophilia in the prognosis of patients with nasopharyngeal carcinoma. Auris Nasus Larynx.

[17] Costello R, O'Callaghan T, Sebahoun G (2005). Eosinophils and antitumour response. Rev Med Interne.

[18] Davis BP, Rothenberg ME (2014). Eosinophils and cancer. Cancer Immunol Res.

[19] Munitz A, Levi-Schaffer F (2004). Eosinophils: 'new' roles for 'old' cells. Allergy.

[20] Molin D (2004). Bystander cells and prognosis in Hodgkin lymphoma. Review based on a doctoral thesis. Ups J Med Sci.

[21] Curran CS, Bertics PJ (2012). Eosinophils in glioblastoma biology. J Neuroinflammation.

[22] Kaur H (2019). Asthma and risk of glioma: a population-based case-control study. BMJ Open.

[23] DPP4 (2019). Inhibition Controls Tumor Growth via Eosinophil Recruitment. Cancer Discov.

[24] Holl EK (2019). Examining peripheral and tumor cellular Immunome in patients with Cancer. Front Immunol.

[25] Giembycz MA, Lindsay MA (1999). Pharmacology of the eosinophil. Pharmacol Rev.

[26] Caruso RA (2011). Ultrastructural descriptions of heterotypic aggregation between eosinophils and tumor cells in human gastric carcinomas. Ultrastruct Pathol.

[27] Kubo H (1999). Cytotoxic properties of eosinophil granule major basic protein for tumor cells. Int Arch Allergy Immunol.

[28] Grauer OM (2008). TLR ligands in the local treatment of established intracerebral murine gliomas. J Immunol.

[29] Curtin JF (2009). HMGB1 mediates endogenous TLR2 activation and brain tumor regression. PLoS Med.

[30] Boix E (2008). The antipathogen activities of eosinophil cationic protein. Curr Pharm Biotechnol.

[31] Nathan CF, Klebanoff SJ (1982). Augmentation of spontaneous macrophage-mediated cytolysis by eosinophil peroxidase. J Exp Med.

[32] Visconti R, Grieco D (2009). New insights on oxidative stress in cancer. Curr Opin Drug Discov Devel.

[33] Sakkal S (2016). Eosinophils in Cancer: Favourable or Unfavourable?. Curr Med Chem.

[34] Rosenberg SA (2001). Progress in human tumour immunology and immunotherapy. Nature.

[35] Carretero R (2015). Eosinophils orchestrate cancer rejection by normalizing tumor vessels and enhancing infiltration of CD8(+) T cells. Nat Immunol.

[36] Simson L (2007). Regulation of carcinogenesis by IL-5 and CCL11: a potential role for eosinophils in tumor immune surveillance. J Immunol.

[37] Wagner LA (2007). EGO, a novel, noncoding RNA gene, regulates eosinophil granule protein transcript expression. Blood.

[38] Xu SP (2015). Downregulation of the long noncoding RNA EGOT correlates with malignant status and poor prognosis in breast cancer. Tumour Biol.

[39] Wu Y (2017). Long noncoding RNA eosinophil granule ontogeny transcript inhibits cell proliferation and migration and promotes cell apoptosis in human glioma. Exp Ther Med.

[40] Sowers JL (2014). The role of inflammation in brain cancer. Adv Exp Med Biol.

[41] Auezova R (2016). Association of preoperative levels of selected blood inflammatory markers with prognosis in gliomas. Onco Targets Ther.

[42] Wang J (2018). Prognostic significance of preoperative neutrophil-to-lymphocyte ratio and platelet-to-lymphocyte ratio in patients with glioma. EXCLI J.

[43] Baecher-Allan C, Anderson DE (2006). Regulatory cells and human cancer. Semin Cancer Biol.

[44] Chongsathidkiet P (2018). Sequestration of T cells in bone marrow in the setting of glioblastoma and other intracranial tumors. Nat Med.

[45] Domenis R (2017). Systemic T cells immunosuppression of Glioma stem cell-derived Exosomes is mediated by Monocytic myeloid-derived suppressor cells. PLoS One.

[46] Reichman H, Karo-Atar D, Munitz A (2016). Emerging roles for Eosinophils in the tumor microenvironment. Trends Cancer.

[47] Zhou XW (2016). Significance of the prognostic nutritional index in patients with glioblastoma: a retrospective study. Clin Neurol Neurosurg.

[48] Zhang H (2019). The predictive value of a preoperative systemic immune-inflammation index and prognostic nutritional index in patients with esophageal squamous cell carcinoma. J Cell Physiol.

[49] Zhong JH, Huang DH, Chen ZY (2017). Prognostic role of systemic immune-inflammation index in solid tumors: a systematic review and meta-analysis. Oncotarget.

[50] Staton CA, Brown NJ, Lewis CE (2003). The role of fibrinogen and related fragments in tumour angiogenesis and metastasis. Expert Opin Biol Ther.

[51] Ostrom QT (2013). CBTRUS statistical report: Primary brain and central nervous system tumors diagnosed in the United States in 2006–2010. Neuro Oncol.

